# Interaction of Catechins with Human Erythrocytes

**DOI:** 10.3390/molecules25061456

**Published:** 2020-03-24

**Authors:** Katarzyna Naparlo, Grzegorz Bartosz, Ireneusz Stefaniuk, Bogumil Cieniek, Miroslaw Soszynski, Izabela Sadowska-Bartosz

**Affiliations:** 1 Department of Analytical Biochemistry, Institute of Food Technology and Nutrition, College of Natural Sciences, Rzeszow University, 35-601 Rzeszow, Poland; katarzyna.naparlo@gmail.com; 2 Department of Bioenergetics and Food Analysis, Institute of Food Technology and Nutrition, College of Natural Sciences, Rzeszow University, 35-601 Rzeszow, Poland; gbartosz@ur.edu.pl; 3 Teaching and Research Center of Microelectronics and Nanotechnology, College of Natural Sciences, University of Rzeszow, 35-959 Rzeszow, Poland; istef@univ.rzeszow.pl (I.S.); cieniek@if.univ.rzeszow.pl (B.C.); 4 Department of Molecular Biophysics, Faculty of Biology and Environmental Protection, University of Lodz, 90-236 Lodz, Poland; miroslaw.soszynski@biol.uni.lodz.pl

**Keywords:** erythrocyte, monometric flavanols, catechin, (−)-epigallocatechin, (−)-epigallocatechin gallate, hemolysis, membrane rigidity, antioxidant

## Abstract

The aim of this study was to characterize the interaction of chosen catechins ((+)-catechin, (−)-epigallocatechin (EGC), and (−)-epigallocatechin gallate (EGCG)) with human erythrocytes and their protective effects against oxidative damage of erythrocytes. Uptake of the catechins by erythrocytes was studied by fluorimetry, their interaction with erythrocyte membrane was probed by changes in erythrocyte osmotic fragility and in membrane fluidity evaluated with spin labels, while protection against oxidative damage was assessed by protection against hemolysis induced by permanganate and protection of erythrocyte membranes against lipid peroxidation and protein thiol group oxidation. Catechin uptake was similar for all the compounds studied. Accumulation of catechins in the erythrocyte membrane was demonstrated by the catechin-induced increase in osmotic resistance and rigidification of the erythrocyte membrane detected by spin labels 5-doxyl stearic acid and 16-doxyl stearic acid. (−)-Epigallocatechin and EGCG inhibited erythrocyte acetylcholinesterase (mixed-type inhibition). Catechins protected erythrocytes against permanganate-induced hemolysis, oxidation of erythrocyte protein thiol groups, as well as membrane lipid peroxidation. These results contribute to the knowledge of the beneficial effects of catechins present in plant-derived food and beverages.

## 1. Introduction

Catechins (monomeric flavanols) and their gallate derivatives are a class of flavonoids mainly present in fruits and vegetables and derived products like fruit juices or jams [1]. Monomeric flavanols are abundant in teas derived from the tea plant *Camellia sinensis*, as well as in some cocoas and chocolates (made from the seeds of *Theobroma cacao*) [2]. Catechins are also present in wine and are found in many other plant species. One gram of dried green tea leaves contain more than 200 mg catechins [3], although total catechin content varies widely depending on species, variety, and growth conditions. The main catechins present in green tea, as well as in black tea, are (−)-epigallocatechin (EGC) and the ester of epigallocatechin and gallic acid, (−)-epigallocatechin gallate (EGCG) [4,5]. (+)-Catechin is present in tea in minor amounts but is the main catechin in broad bean, black as well as white grape, plum, rhubarb, and raisins [6]. The food sources of catechins (tea, chocolate, apples, pears, grapes, and red wine) are very popular and highly consumed. Tea catechins consumed by volunteers at a total dose of 240 mg have been found to reach a concentration of up to 0.5 μM in blood plasma [7]. Consumption of eight cups of black tea every 2 h daily by volunteers has brought their blood plasma catechin level up to 1 μM [8].

As the consumption of catechins is significant, these compounds raise considerable interest with respect to their health effects. Numerous beneficial health effects of catechins have been demonstrated. These compounds have been claimed to reduce cardiovascular disease mortality by inhibiting endothelial dysfunction [9], to disrupt inflammation mediated by lipid and cholesterol oxidation [10], prevent onset and complications of diabetes [11], as well as prevent and slow down the development of metabolic syndrome [12]. They are thought to exert renoprotective actions that may be of importance in diseases, such as glomerulonephritis, diabetic nephropathy, and chemically-induced kidney insufficiency [13], have antihypertensive and neuroprotective actions [14] and improve exercise performance and recovery [15]. The action of catechins is mainly, though by no means exclusively, ascribed to their direct or indirect antioxidant activities. Previously, we demonstrated excellent antioxidant properties of selected catechins to compare with several other natural and synthetic compounds and related to glutathione and ascorbate as key endogenous antioxidants [16].

The effects of catechins are critically dependent on their cellular action. We studied the protective effect of (+)-catechin and (−)-epigallocatechin gallate on the yeast *Saccharomyces cerevisiae* with respect to cellular mortality dependent on oxidative stress [17]. There are reports on the effects of catechins on erythrocytes. (+)-Catechin has been found to protect human erythrocytes against pentachlorophenol-induced oxidative damage [18]. Tea catechins have been demonstrated to show significant protection to erythrocyte against oxidative stress induced by *tert*-butyl hydroperoxide. The effect is more pronounced in the older age group compared to the lower age group [19]. We demonstrated that selected monomeric catechins, including (+)-catechin and EGCG, protected erythrocytes against oxidative hemolysis induced by 2,2′-azobis(2-amidinopropane) dihydrochloride (AAPH) and hypochlorite [16]. Protection against hypochlorite-induced hemolysis has also been confirmed by others [20]. Black tea extract is found to protect erythrocytes against damage induced by γ-radiation [21]. EGCG has protected erythrocyte membrane Ca^2+^-ATPase and (Na^+^, K^+^)-ATPase against inactivation induced by *tert*-butyl hydroperoxide [22], protected erythrocytes against oxidative effects of cigarette smoke [23], and partly prevented hemolysis induced by cyclosporine [24]. (+)-Catechin has protected erythrocyte membranes against lipid peroxidation induced by hydrogen peroxide [25] and erythrocytes against hemolysis induced by cumene hydroperoxide [26]. Catechins, including EGCG and (+)-catechin, among other flavonoids, have been reported to increase the activity of a plasma membrane redox system (PMRS) that transfers electrons from intracellular substrates to extracellular electron acceptors by entering erythrocytes and donating electrons to PMRS [27].

However, EGCG and other catechins have been found to inhibit catalase in a cell-free system and in K562 cells, thus contributing to oxidative stress and an increase in the intracellular level of reactive oxygen species [28]. Prooxidative effects of tea catechins, including EGCG (decrease in the level of reduced glutathione, increase in the ratio of oxidized/reduced glutathione, and elevation in methemoglobin level), have been observed in glucose-6-phosphate dehydrogenase-deficient erythrocytes, leading to a recommendation against excessive consumption of concentrated tea polyphenolic products by glucose-6-phosphate dehydrogenase-deficient subjects [29]. 

**Figure 1:**
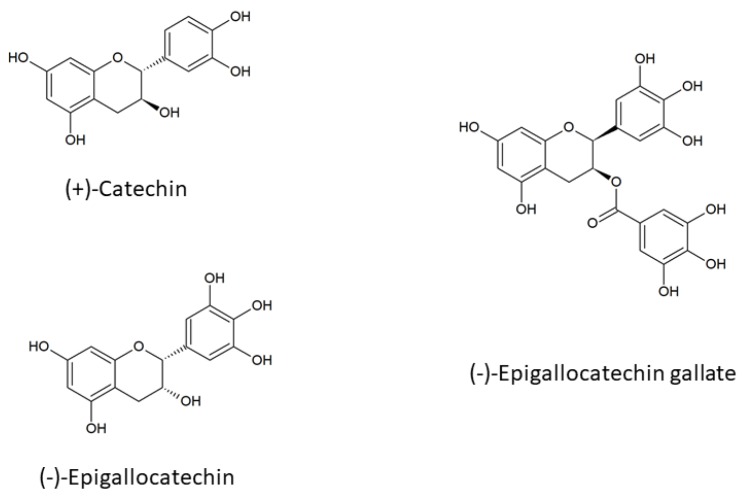
Structures of monomeric flavanols used in this study.

The aim of this study was to further characterize the interaction of chosen catechins (Figure 1) with model human cells, viz. erythrocytes, which are considered a prime target for oxidant attack due, i.e., to their function as oxygen carriers and the presence of high contents of polyunsaturated fatty acid in their membranes. These simple cells allow for easy studies of the effects of xenobiotics on the cellular membrane, studies of their transport, and examination of antioxidant effects not complicated by the intermediacy of cellular effects dependent on protein synthesis de novo. Erythrocytes interact with xenobiotics circulating in blood after ingestion in the intestine. We wanted to quantify and compare the uptake of catechins by human erythrocytes, which, according to our best knowledge, has not been studied quantitatively. Due to their hydrophobicity, catechins can be expected to accumulate partly in the erythrocyte membrane; if so, a decrease in osmotic fragility of erythrocytes should be expected due to membrane expansion, and we intended to verify this expectation. Moreover, the accumulation of catechins in the membrane should affect membrane fluidity, and this effect was checked by EPR spectroscopy using fatty acid spin probes. Although antioxidant effects of flavonoids on erythrocytes have already been studied, we wanted to provide additional data in this respect by studying the protection of erythrocytes against oxidative hemolysis induced by potassium permanganate and protection of erythrocyte membranes against lipid peroxidation and protein thiol oxidation.

## 2. Results

### 2.1. Catechin Uptake by Erythrocytes

**Figure 2:**
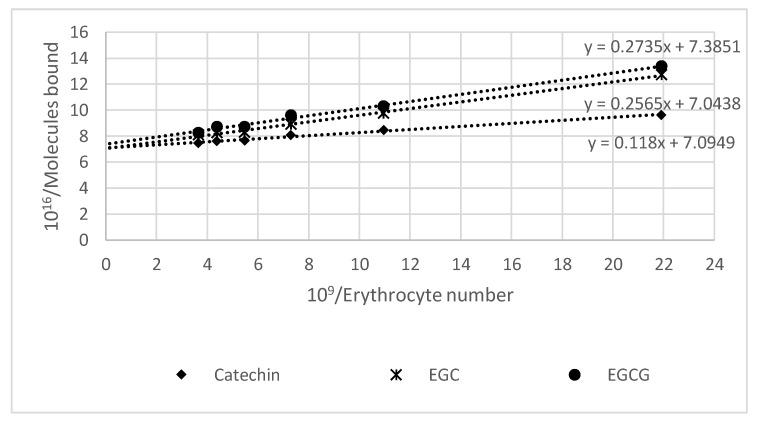
The double reciprocal plot of the uptake of selected flavanols by human erythrocytes. EGC, epigallocatechin; EGCG, epigallocatechin gallate.

Incubation of erythrocytes with catechins resulted in the uptake of these compounds by the cells. The study of the dependence of the amount of catechins taken up on the amount of erythrocytes showed a saturation behavior. Presentation of the data in the form of a double reciprocal plot of dependence of the number of molecules of monomeric flavanols taken up on the number of erythrocytes (Figure 2) demonstrated similar uptake of all compounds tested. The number of molecules of the studied compounds taken up by 2.5 × 10^8^ erythrocytes from 500 μL of 50 μM solutions of monomeric flavanols (these values correspond to erythrocyte concentration in the blood) was (1.19 ± 0.03) × 10^6^ for catechin, (1.23 ± 0.21) × 10^6^ for epigallocatechin (EGC), and (1.18 ± 0.14) × 10^6^ for EGCG.

### 2.2. Effect of Selected Flavanols on Osmotic Fragility of Erythrocytes

From osmotic fragility curves, the values of NaCl concentration evoking 50% hemolysis (c_50_) were determined (Figure S1). When erythrocytes (hematocrit of 10%) were incubated with 50 μM selected flavanols at 37 °C for 90 min, catechins increased erythrocyte’s osmotic fragility, as evidenced by an increase in c_50_ values. Nevertheless, when catechins were added to erythrocytes, and osmotic fragility was assessed immediately (the contact of catechins with erythrocytes was about 5 min at room temperature), these compounds protected erythrocytes against osmotic hemolysis, as evidenced by decreased values of c_50_ (Table 1).

### 2.3. Effect of Selected Catechins on Membrane Fluidity

Examples of EPR spectra of 5-doxyl stearic acid (5DS) and 16-doxyl stearic acid (16NS) embedded in erythrocyte membranes in the absence and in the presence of EGCG are shown in Figure S2. The catechins had generally a tendency to increase the rotational correlation time τ_c_ of 16DS (Table 2) and order parameter (S) (Table 3) of both probes embedded in erythrocyte membrane lipids.

### 2.4. Effect of Catechins on Membrane Acetylcholinesterase

**Figure 3:**
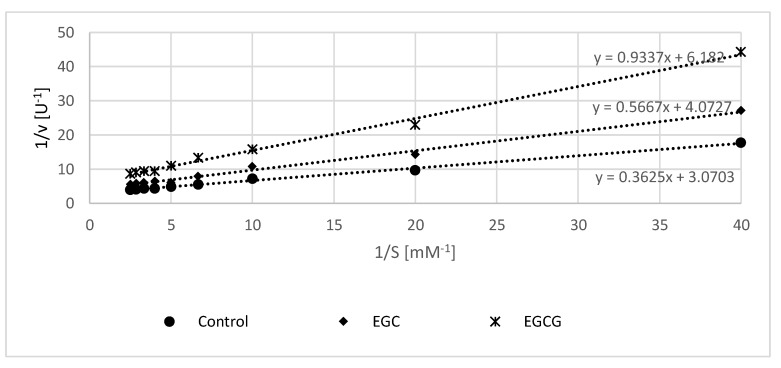
Lineweaver–Burk plot of erythrocyte membrane acetylcholinesterase activity in the absence and in the presence of 50 μM (−)-epigallocatechin (EGC) and 50 μM (−)-epigallocatechin gallate (EGCG).

Catechin at reasonable concentrations (up to 50 μM) did not have any discernible effect on the activity of erythrocyte membrane acetylcholinesterase (not shown). EGC and EGCG inhibited the enzyme in a concentration-dependent manner, evoking a ca 30% and 35% inhibition, respectively, at a concentration of 50 μM. Lineweaver–Burk plot of inhibition of acetylcholinesterase by 50 μM EGC and EGCG pointed to a mixed type of inhibition in both cases (Figure 3, Table 4). 

### 2.5. Protection against Oxidative Hemolysis

We chose the turbidimetric method of monitoring hemolysis, which, although being less precise than the approach based on the centrifugation of erythrocyte suspensions and measurement of released hemoglobin, is much simpler, can be executed in a microplate reader, and is satisfactory for comparative purposes.

**Figure 4:**
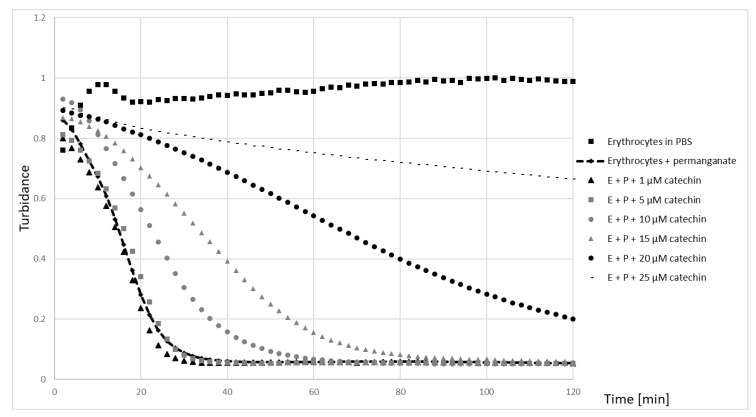
The exemplary curve of permanganate-induced hemolysis in the presence of various concentrations of catechin. E—erythrocytes; P—permanganate.

**Figure 5:**
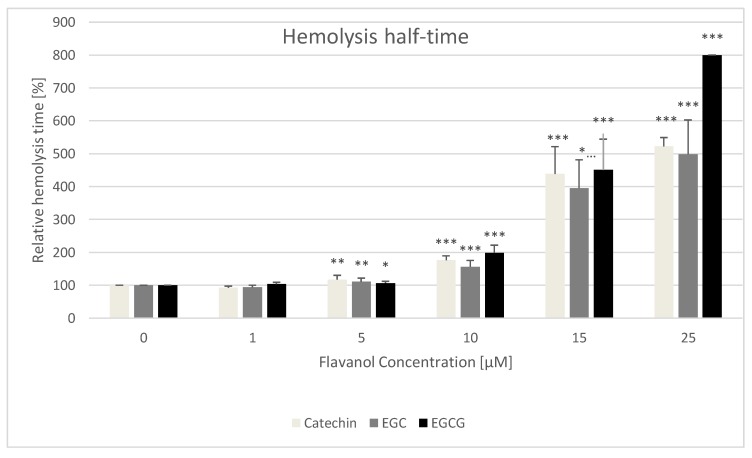
Effect of monomeric flavanols on the relative hemolysis half-time of erythrocytes. Half-time of hemolysis of control samples assumed as 100%. * *p* < 0.05, ** *p* < 0.01, *** *p* < 0.001 (with respect to control).

**Figure 6:**
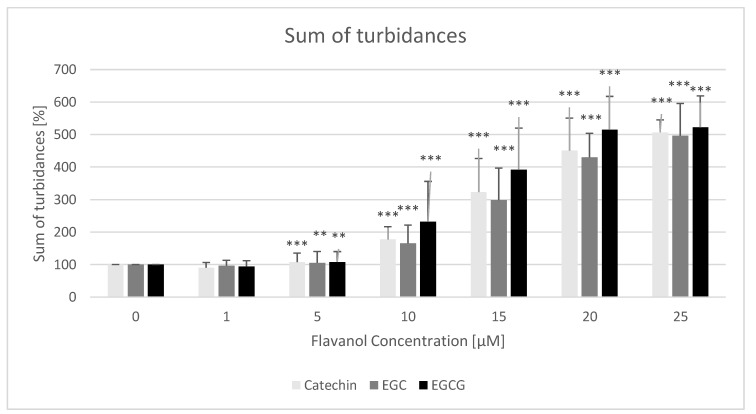
Effect of monomeric flavanols on the hemolysis of erythrocytes estimated from the sum of turbidance values during 120-min measurements (every 2 min). * *p* < 0.05, ** *p* < 0.01, *** *p* < 0.001 (with respect to control). Values for control samples were assumed as 100%.

An example of the time course of turbidity of erythrocyte suspensions subjected to the action of 100 μM potassium permanganate in the presence of various concentrations of catechin is shown in Figure 4. Hemolysis of half-time (time corresponding to a decrease of turbidance to 50% of the initial values) in the absence of studied compounds was 19.9 ± 1.9 min. Catechins increased the time necessary to reach 50% hemolysis (Figure 5). Another means of quantifying the extent of hemolysis was the summation of subsequent turbidance values during 2-h measurements. Also, this parameter demonstrated the protective effect of catechins (Figure 6). 

Microscopic examination of erythrocytes subjected to the action of permanganate showed complete hemolysis of cells in the absence of studied flavanols after >20 min (Figure S3b) and protection from hemolysis by selected flavanols. Interestingly, while initially erythrocytes treated with monomeric flavanols showed echinocytosis (Figure S3e), later on, this echinocytosis disappeared (Figure S3f), apparently due to oxidation of the compounds studied by peroxymanganate and weaker interaction of oxidation products of monomeric flavanols with erythrocyte membranes. 

### 2.6. Protection against Oxidation of Erythrocyte Membrane Components

All the compounds studied tested dose-dependently protected against hypochlorite-induced oxidation of membrane protein thiol groups (Table 5) and lipid peroxidation (Table 6).

## 3. Discussion

The present study was aimed at the characterization of the interaction of three chosen catechins with the human erythrocyte and the protective effect of the catechins against oxidant-induced damage. Erythrocytes are a main component of blood, and ingested catechins can interact with these cells. Moreover, erythrocytes are a simple and convenient model to study the cellular effects of catechins, especially effects on the plasma membrane. 

The catechins studied were bound by erythrocytes at similar amounts (Figure 1), corresponding to about 1.12 × 10^6^ molecules, when 2.5 × 10^8^ erythrocytes interacted with 50 μM solutions of monomeric flavanols in a total volume of 500 μL (this erythrocyte concentration corresponds roughly to that in the blood). Such a high concentration of catechins is not attainable in vivo; nevertheless, these model conditions allow for an easy examination of the effect of monomeric flavanols on erythrocytes, while fractions of these effects can take place in vivo. 

Due to their considerable hydrophobicity, catechins are expected to accumulate predominantly in the plasma membrane of the erythrocyte. Scanning electron microscopy observations showed that EGCG induced morphological alterations in human erythrocytes from their normal discoid form to crenated echinocytes. We confirmed this observation also for other catechins (Figure S3). According to the bilayer couple hypothesis [30], the shape changes induced in human erythrocytes by foreign molecules are due to differential expansion of the two monolayers of the red blood cell membrane. When the exogenous molecules locate into the outer membrane leaflet, echinocytosis occurs. NMR studies have shown that EGCG molecules are preferably located in regions near to the surface of the lipid bilayer with their B-ring and the galloyl moiety at the level of the trimethylammonium groups of phosphatidylcholines [31]. 

Accumulation of catechins in the erythrocyte membrane should lead to membrane expansion and an increase in the osmotic resistance of erythrocytes. According to the Ponder’s classical description of hemolysis [32], erythrocytes (which in isotonic solutions have a surface excess with respect to the spherical shape) swell in hypotonic solutions until attaining a spherical shape. Since the erythrocyte membrane is essentially inextensible, hemolysis then occurs. If the membrane surface is augmented (e.g., by incorporation of exogenous substances), a spherical shape is attained at a lower osmolarity of the medium, i.e., erythrocyte osmotic fragility is decreased. The protection of erythrocytes against osmotic hemolysis by green tea extract and EGCG has been reported [33]. We confirmed this observation for all catechins studied (Table 1). 

Interestingly, the protective effect of catechins on erythrocyte osmotic fragility was observed only under conditions of short (up to 5 min) incubation of catechins with erythrocytes. Prolonged incubation (90 min at 37 °C) evoked a reverse effect, viz. increase in osmotic fragility of erythrocytes by the catechins studied. It is a surprising effect, pointing to the cautious design of experiments and the interpretation of experimental results. We could suggest two reasons for an explanation of this effect (i) during prolonged incubation, more and more catechins are taken up by erythrocytes, which may lead to increase in the osmolarity of cell interior and thus easier hemolysis in hypotonic solutions due to a greater difference in osmolarity between cell interior and exterior; however, this effect does not seem significant at low concentration of catechins employed; (ii) as demonstrated previously, catechins generate hydrogen peroxide during prolonged incubation [34,35]. Hydrogen peroxide may damage the erythrocyte membrane and increase its fragility, including osmotic fragility. 

Accumulation of exogenous compounds in the erythrocyte membrane can lead to changes in the ordering of membrane lipids. We observed generally an increase in the rotational correlation time of 16DS (Table 2) and order parameter of membrane lipids (Table 3), estimated with two spin labels—5DS and 16DS. The use of these spin probes is complicated by the fact that pK_a_ values of fatty acid spin probes are in the range of near-neutral pH, which leads to the appearance of two components in their EPR spectra, corresponding to non-ionized and ionized molecules [36,37]. However, the resolution of both components is not easy in standard EPR spectra, so we restricted our analysis to the dominant component, corresponding to non-ionized probes (Figure S2). Both probes evidenced an increase in the rigidity of the hydrophobic core of the membrane. Our results pointed to the rigidification of membrane lipids at high catechin concentrations, not attainable in vivo. Apparently, such concentrations were saturated with respect to the effects of catechins on membrane fluidity, which could explain the lack of concentration dependence of the effect. Nevertheless, other reports document membrane rigidification by monomeric flavanols also at their lower concentrations. EGCG-induced increase in erythrocyte membrane anisotropy measured with DPH, known to be located in the hydrophobic zone of the erythrocyte, has been found [33]. In isolated human erythrocyte membranes, EGCG has induced a moderate increase in laurdan general polarization, a result that implies a decrease in the molecular dynamics at the hydrophobic–hydrophilic interphase, which indicates an increase in the rigidity of this region of the membrane [20]. The rigidifying effect of catechins can contribute to their antioxidant effect. As initiation and propagation of free radical reactions depend on the mobility of membrane components, increased membrane rigidity hampers lipid peroxidation [38,39].

Inhibition of acetylcholinesterase by natural compounds, including polyphenols, is of interest as acetylcholine deficit accompanies Alzheimer’s disease (AD). During the progression of AD, many different types of neurons deteriorate, although there is a profound loss of forebrain cholinergic neurons, accompanied by a progressive decline in acetylcholine, and acetylcholinesterase inhibitors provide effective temporary relief of symptoms at least in some patients [40]. Although the function of acetylcholinesterase on the erythrocyte surface remains a mystery, this membrane-bound enzyme is very useful for model studies of acetylcholinesterase inhibitors. Inhibition of erythrocyte membrane acetylcholinesterase by EGCG has been reported [41,42], although the kinetic aspects of this inhibition have not been reported to our best knowledge. Our results pointed to a mixed-type inhibition of erythrocyte membrane acetylcholinesterase activity by EGC and EGCG. 

An association between the consumption of tea, rich in catechins, and the reduced risk of AD have been reported. Tea has been suggested to have anti-amyloid effects [43] and ameliorate cognitive dysfunction [44]. These effects have been mainly ascribed to the antioxidant and anti-inflammatory effects of tea catechins, but the inhibition of acetylcholinesterase by EGCG may contribute to this effect. The EGCG concentration attainable in the blood is low, but the octanol/PBS partition coefficient of this compound is about 26 [45], so it can reach much higher concentrations in the membranes where acetylcholinesterase is located. 

We found a protective effect of all catechins studied on the hemolysis induced by potassium permanganate (Figure 3, Figure 4 and Figure 5). Potassium permanganate is a strong oxidizing agent that can induce hemolysis and methemoglobinemia [46,47]. Protection by catechins against other oxidants inducing hemolysis has been reported previously [16,20,24]. Catechins protected also against oxidation of erythrocyte membrane protein thiol groups and membrane lipid peroxidation. In all cases, the effects of three catechins studied were similar, speaking against a significant contribution of the gallate group of EGCG to the antioxidant properties of this compound. 

The parameters of membrane oxidative damage studied (lipid peroxidation and membrane thiols) are typically used in studies of oxidative damage and antioxidant effects. In these experiments, we used hypochlorite, which is a physiologically relevant oxidant. HOCl is generated by the reaction of H_2_O_2_ with chloride ions (Cl^−^) catalyzed by myeloperoxidase. Up to 80% of the H_2_O_2_ generated by activated neutrophils may be used to produce local concentrations as high as 20–400 μM HOCl within an hour [48]. Relatively high concentrations of the catechins were used in order to observe the significant effects of membrane protection. However, other parameters may be more sensitive to demonstrate the protective effects of lower antioxidant concentrations. One such parameter is the rate of Band 3-mediated sulfate transport, which has been demonstrated to be affected by hydrogen peroxide under conditions inducing neither lipid peroxidation nor thiol oxidation [49].

## 4. Materials and Methods 

### 4.1. Chemicals and Equipment

Dimethyl sulfoxide (DMSO; DMS555.500, Purity: ≥ 99.9%), sodium phosphate monobasic (SPM306.500; purity ≥ 98–103%), sodium phosphate dibasic (SPD579.500; purity ≥ 98–102%), as well as phosphate-buffered saline (PBS tablets; PBS404.200), produced by BioShop Canada Inc. (Burlington, Ontario, Canada), were purchased from Lab Empire (Rzeszow, Poland). Sodium chloride (NaCl; 31434, purity ≥ 99.5%), produced by Honeywell Speciality Chemicals (Seelze, Germany), was purchased from Alchem (Rzeszow, Poland). Sodium hypochlorite (1.05614) was purchased from Merck (Darmstadt, Germany). Trichloroacetic acid (TCA; 115779700, purity ≥ 98%) was purchased from Chempur (Piekary Slaskie, Poland). 2-Thiobarbituric acid (TBA) was purchased from Serva Electrophoresis GmbH (Heidelberg, Germany). TBA was dissolved in 0.1 M NaOH at a concentration of 0.67%. Spin probes [5-doxyl stearic acid (5DS) and 16-doxyl stearic acid (16DS)] and all other reagents, if not stated otherwise, were purchased from Sigma-Alrich (Poznan, Poland) and were of analytical grade. Distilled water was purified using a Milli-Q system (Millipore, Bedford, MA, USA).

Fluorometric and absorptiometric measurements were done in a Spark multimode microplate reader (Tecan Group Ltd., Männedorf, Switzerland). All measurements were performed in triplicate and repeated at least three times on different preparations. (+)-Catechin (C; 43412, purity ≥ 99%), (−)-Epigallocatechin gallate (EGCG; E4143, purity ≥ 95%) and (−)-Epigallocatechin (EGC; E3768, purity ≥ 95%) were purchased from Sigma-Aldrich (Poznan, Poland). They were dissolved in PBS.

### 4.2. Ethical Approval 

The study was approved by the Bioethics Committee of the University of Lodz (Permit No. KBBN-UŁ/I/3/2013).

### 4.3. Preparation of Erythrocytes

Eight milliliters of peripheral blood from healthy donors (lab volunteers) were collected in EDTA tubes and used within the day of its collection. Erythrocytes were isolated by centrifugation for 10 min at 3000 rpm at 4 °C. The plasma and buffy coat were removed by aspiration. The red blood cells (RBCs) were washed four times with ice-cold PBS. Washed RBCs were suspended at various hematocrit from 4% to 60%.

### 4.4. Preparation of Erythrocyte Ghosts

Erythrocyte ghosts were prepared from washed erythrocytes according to the method of Dodge et al. [50] with some modifications. Briefly, after incubation, erythrocytes were hemolyzed on ice with 20 volumes of 20 mM phosphate buffer, pH 7.4, containing 1 mM ethylenediaminetetraacetic acid (EDTA) and centrifuged at 4 °C at 20,000× *g* for 20 min. The ghosts were resuspended in ice-cold 10 mM and then 5 mM phosphate buffer, pH 7.4 containing 1 mM EDTA, centrifuged, and this process was continued until the ghosts were free from residual hemoglobin. Finally, the erythrocyte ghosts were resuspended in 20 mM phosphate buffer, pH 7.4. The protein concentration was estimated by the method of Lowry et al. [51].

### 4.5. Transport of Catechins into Erythrocytes

The solution of a selected flavanol was incubated with different volumes of erythrocytes, and, after centrifugation, the catechin concentration in the supernatant was determined by measurements of fluorescence of the monomeric flavanol. 

#### 4.5.1. Determination of Optimal Excitation and Emission Wavelengths of Catechins

The optimal excitation and emission wavelengths for 50 µM C, EGCG, as well as EGC, were determined. The standard curve was prepared to determine the concentration dependence of the fluorescence of a studied compound.

#### 4.5.2. Uptake of Catechins by Human Erythrocytes

A series of erythrocytes suspensions in PBS were prepared (total volume 450 µL), and 50 µL of the solution of a selected compound (catechin, EGC, or EGCG) in PBS was added to obtain a final catechin concentration of 50 µM. Then, the samples were mixed and incubated for 1 h at room temperature (22–25 °C) with constant mixing. Finally, the samples were centrifuged (10 min, 5000 rpm), and the fluorescence was measured for 100 µL of selected supernatant mixed with 100 µL of DMSO (excitation 230 nm, emission 290 nm). The amount of catechins taken up was dependent on the amount of erythrocytes, and the reciprocal plots (plots of the (1/(number of molecules bound) as a function of 1/(erythrocyte number)) were linear. They were presented as 10^16^/(number of molecules bound by erythrocytes in the sample) vs. 10^9^/(number of erythrocytes).

### 4.6. Effect of (+)-Catechin, (−)-Epigallocatechin, and (−)-Epigallocatechin Gallate on the Osmotic Fragility of Human Erythrocytes

Washed RBCs were suspended at 10% hematocrit and added with catechins to a final concentration of 50 µM. Immediately or after 90-min incubation at 37 °C, 50 µL of erythrocyte suspensions were added to 950 µL solutions of various NaCl concentrations (0.34, 0.35, 0.36, 0.37, 0.38, 0.39, 0.40, 0.41, 0.43, 0.44, and 0.45%). The control sample consisted of 50 µL erythrocyte suspension and 950 µL of water. Immediately, the samples were centrifuged (5 min, 5000 rpm), and the absorbance of the supernatant was measured (540 nm). From the osmotic fragility curves, NaCl concentration causing 50% hemolysis (c_50_) was determined. 

### 4.7. Potassium Permanganate (KMnO_4_)-Induced Hemolysis

Aliquots of erythrocyte suspensions in PBS were mixed with selected catechin at a final concentration range of 1–25 μM (final volume of 200 μL) and incubated for 15 min with shaking at 37 °C. Then, 0.1 mM potassium permanganate (final concentration), as optimal concentration to induce hemolysis, was added, and turbidance (700 nm) was measured every 2 min for 120 min, with intermittent shaking. 

For all determinations, hemolysis time (seconds) and relative hemolysis time with respect to that of control erythrocytes, assumed as 100%, were calculated as 100% × (time (seconds) for test compound)/(mean time (seconds) for control sample containing erythrocytes and KMnO_4_ only) (Figure 5). As an alternative measure of the extent of hemolysis, the sums of turbidances of erythrocyte suspensions measured at 2-min intervals were calculated. Again, the value for control erythrocytes was assumed as 100%, and appropriate values for samples supplemented with catechins were expressed as a percent of this value (Figure 6).

### 4.8. Estimation of Acetylcholinesterase (AChE) Activity 

The activity of AChE was measured using the colorimetric method described by Ellman et al. [52] with slight modifications, employing acetylthiocholine iodide (AcTCh) as a substrate. Briefly, 5 µL of erythrocyte membranes were added to 0, 1, 2, 3, 5, 10, 20, 30 µM, or 50 µM (+)-catechin, (−)-epigallocatechin, or (−)-epigallocatechin gallate solutions with 0.1 M phosphate buffer pH 7.4, 0.5 mM 5,5-dithio-bis-(2-nitrobenzoic acid) (DTNB, Ellman reagent) (10 µL of 10 mM DTNB stock), as well as 0.5 mM acetylthiocholine iodide (20 µL of 5 mM acetylthiocholine iodide stock) (the final volume: 200 µL). The absorbance was measured in a plate reader, every 10 s for 3 min, at 412 nm. The membrane protein concentration in the assay mixture was 67 μg/mL. 

Kinetics of inhibition of acetylcholinesterase was also estimated. Two measuring series (one with phosphate buffer, pH 7.4, Ellman reagent, different amounts of the substrate, and 50 μM selected flavanol (final volume 200 µL), another without the flavanol) were added to wells of a 96-well plate, and time course of the increase in absorbance at 412 nm was measured. Acetylcholinesterase activity was calculated, and based on the obtained average values, a Lineweaver–Burk graph ((1/(velocity of enzymatic reaction) vs. 1/(substrate concentration)) in the absence and in the presence of the studied compounds was made (Figure 2), and the type of inhibition was determined.

### 4.9. Assessment of Membrane Fluidity 

Briefly, 200 µL aliquots of erythrocyte membranes (2.7 mg protein/mL) were added with 10 mM catechins to desired concentrations (0, 50, 100, and 250 µM). Two microliters of selected 10 mM probes (5DS or 16DS) in DMSO were added to each sample, mixed, and after 10 minutes of incubation, electron paramagnetic resonance (EPR) measurements were performed using microhematocrit capillaries (non-heparinized microhematocrit tubes ∼75 μL; 1.55 × 75 mm; Medlab Products, Raszyn, Poland) in a BRUKER multifrequency and multi resonance FT-EPR ELEXSYS E580 apparatus (BRUKER BIOSPIN, Billerica, MA, USA) [X-band(~9.5GHz) CW-EPR spectrometer consisting of an ER4119-HS cavity]. Sample capillaries were placed into a quartz EPR sample tube and centered in a microwave cavity. The following settings were used: central field, 3353 G; modulation amplitude, 1 G; modulation frequency, 100 kHz; microwave power, 23.77 mW; power attenuation 2 dB; scan range, 100 G; conversion time, 25 ms; sweep time, 25.6 s. The spectra were recorded in 1024 channels, and the number of accumulated scans was 3. The EPR spectra were recorded and analyzed using the Xepr 2.6b.74 software. Xepr is a comprehensive software package of the ELEXSYS series, accommodating the needs of every user with highly developed acquisition and processing tools. 

Rotational correlation time τ_c_ was calculated according to Schreier et al. [53]:

(1) $$\tau_{c}=\frac{1}{2\;}\kappa W_{0\;}\left(\sqrt{\frac{h_{0}}{h_{+1}}}+\sqrt{\frac{h_{0}}{h_{-1}}}-2\right)$$

where τ_c_—time when the spin probe undergoes full rotation, κ- constant equal to 1.19 × 10^−9^ s, W_0_—width of the mid-line spectrum, h_0_—the height of the mid-line spectrum, h_+1_—the height of the low-field line of the spectrum, h_−1_—the height of the high-field line of the spectrum.

Order parameter (S) was calculated according to Hubbell and McConnell [54]:

(2) $$S=\frac{2\;A_{║}-2A_{┴}}{2\;\left[A_{zz}-\left(A_{xx}+A_{yy}\right)/2\right]},$$

where 2 A_║_and 2 A_┴_ are the separations between the outer and inner extrema, respectively, in the experimental spectrum, and A_xx_, A_yy_, and A_zz_ are the values of the principal components of the hyperfine tensor (A_xx_ = A_yy_ = 6 G, A_zz_ = 32 G) [53].

### 4.10. Estimation of the Protective Effects of Catechins on Erythrocyte Membrane Protein Thiol Groups

Erythrocyte membranes (1 mg protein/mL) in 0.1 M sodium phosphate buffer, pH 7.4, were treated with 500 μM (final concentration) sodium hypochlorite for 30 min, in the absence or in the presence of various concentrations of the compounds studied. Then, 100 μL aliquots of membrane suspension were added to wells of a 96-well microplate, followed by 50 μL of 10% solution of sodium dodecyl sulfate (SDS), 50 μL of 0.2 M phosphate buffer, pH 8, and 10 μL of 10 mM DTNB solution. After 15-min incubation in the dark, absorbance was measured at 412 nm. Corrections were made for the absorbance of DTNB solution and absorbance of solutions of the compounds studied. Percent protection was calculated as 100% × ((amount of thiol groups in a sample treated with NaOCl and a given concentration of catechin) − (amount of thiol groups in a sample treated with NaOCl in the absence of any protective agent))/((amount of thiol groups in a control sample) – (amount of thiol groups in a sample treated with NaOCl in the absence of any protective agent)) (Table 5).

### 4.11. Estimation of the Protective Effects of Catechins on Erythrocyte Membrane Lipid Peroxidation

Erythrocyte membranes were treated as above. Then, 200 μL aliquots of membrane suspensions were pipetted to Eppendorf tubes, followed by 250 μL of cold 10% trichloroacetic acid and 250 μL of 0.67% thiobarbituric acid in 0.1 M NaOH. The tubes were heated at 100 °C for 20 min, cooled, centrifuged, and the absorbance of the supernatants was measured at 532 nm. Percent protection was calculated as 100% × ((amount of lipid peroxidation products in a sample treated with NaOCl and a given concentration of catechin) − (amount of lipid peroxidation products in a sample treated with NaOCl in the absence of any protective agent))/((amount of lipid peroxidation products in a control sample) − (amount of lipid peroxidation products in a sample treated with NaOCl in the absence of any protective agent)) (Table 6).

### 4.12. Statistical Analysis

The error bars are standard deviation. The paired Student’s t-test was performed to estimate the differences between samples and control. Kruskal–Wallis test was also performed to determine differences between antioxidant-treated and non-treated cells. *p* ≤ 0.05 was considered as statistically significant. Statistical analysis of the data was performed using the STATISTICA software package (version 13.3, StatSoft Inc. 2016, Tulsa, OK, USA).

## 5. Conclusions

The presented results characterized quantitatively the interaction of catechins with human erythrocytes, showing the similarity of the interaction of catechin, EGC, and EGCG, confirmed their rigidifying effect on erythrocyte membrane, demonstrated and, characterized inhibition of erythrocyte acetylcholinesterase activity by EGC and EGCG, and extended the knowledge on the antioxidant effects of catechins on the erythrocyte as well as erythrocyte membranes. Antioxidant effects of catechins on the erythrocyte shown contribute to the understanding of the beneficial effects of catechins present in plant-derived food and beverages on human health.

## Figures and Tables

**Table 1:** Effect of selected catechins on NaCl concentration causing 50% hemolysis (c_50_) of erythrocytes (mM). Mean ± SD, *n* = 3.

Compound	c_50_ (mM) (Measurement after Incubation (37 °C, 90 min))	c_50_ (mM) (Measurement Asap)
Control	65.4 ± 0.4	65.2 ± 0.8
C	66.5 ± 0.5 *	62.8 ± 0.8 *
Epigallocatechin (EGC	67.0 ± 0.7 *	63.5 ± 0.7 *
Epigallocatechin gallate (EGCG)	66.1 ± 0.4 *	62.7 ± 0.8 **

* *p* < 0.05, ** *p* < 0.01.

**Table 2:** Effect of catechins on the rotational correlation time (in nanoseconds) of 16-doxyl-stearic acid in erythrocyte membranes. Mean values ± SD, *n* ≥ 3.

Compound	Rotational Correlation Time τ_c_ (ns)		
Concentration (μM)	Catechin	EGC	EGCG
0	1.73 ± 0.03		
50	1.62 ± 0.08	1.78 ± 0.005	1.77 ± 0.10
100	1.73 ± 0.28	1.86 ± 0.03 **	1.83 ± 0.20
250	1.81 ± 0.15	1.99 ± 0.23	1.79 ± 0.01 *

* *p* < 0.05, ** *p* < 0.01.

**Table 3:** Effect of catechins on the order parameter of 5-doxyl stearic acid (5DS) and 16-doxyl-stearic acid (16DS) in erythrocyte membranes. Mean values ± SD, *n ≥* 3.

**5DS**			
**Compound**	**Order Parameter S**		
**Concentration (μM)**	**Catechin**	**EGC**	**EGCG**
0	0.610 ± 0.006		
50	0.616 ± 0.007	0.616 ± 0.007	0.616 ± 0.007
100	0.617 ± 0.012	0.617 ± 0.012	0.617 ± 0.012
250	0.618 ± 0.008	0.618 ± 0.008	0.618 ± 0.008
**16DS**			
**Compound**	**S**		
**Concentration (μM)**	**Catechin**	**EGC**	**EGCG**
0	0.145 ± 0.001		
50	0.150 ± 0.002 **	0.148 ± 0.003	0.147 ± 0.001 *
100	0.152 ± 0.003 **	0.150 ± 0.004 *	0.147 ± 0.002
250	0.153 ± 0.002 ***	0.156 ± 0.010 *	0.150 ± 0.002 **

Note: * *p* < 0.05, ** *p* < 0.01, *** *p* < 0.001

**Table 4:** Effect of EGCG on the kinetic parameters of erythrocyte membrane acetylcholinesterase. Mean values ± SD, *n* ≥ 3.

	Michaelis Constant K_m_ (μM)	Maximal Velocity V_m_ (U/g Protein)
Control	118 ± 9	4.79 ± 0.34
+50 μM EGC	139 ± 16	3.66 ± 0.45 *
+50 μM EGCG	151 ± 25 *	2.41 ± 0. 28 ***^♣♣^

* *p* < 0.05, *** *p* < 0.001 with respect to catechin, ^♣♣^ *p* < 0.01 with respect to ECG.

**Table 5:** Protection by monomeric flavanols against hypochlorite-induced oxidation of erythrocyte membrane protein thiol groups. * *p* < 0.05, ** *p* < 0.01 (with respect to control).

Compound	Catechin	EGC	EGCG
0.1	10.3 ± 1.1 *	11.1 ± 1.9 *	13.6 ± 1.2 *
0.2	16.7 ± 3.4 *	17.7 ± 2.1 *	18.2 ± 2.0 *
0.5	32.8 ± 3.8 *	28.3 ± 3.4 *	31.5 ± 8.6 *
1	58.4 ± 13.5 **	44.6 ± 9.9 **	50.2 ± 5.7 *

**Table 6:** Protection by monomeric flavanols against hypochlorite-induced erythrocyte membrane lipid peroxidation. * *p* < 0.05, ** *p* < 0.01 (with respect to control).

Compound	Catechin	EGC	EGCG
0.1	19.0 ± 2.1 *	16.8 ± 1.3 *	24.8 ± 2.0 *
0.2	36.1 ± 5.7 *	27.7 ± 2.2 *	41.1 ± 1.9 *
0.5	44.7 ± 1.5 *	37.4 ± 2.6 *	46.8 ± 3.2 *
1	60.6 ± 2.0 *	59.7 ± 1.5 **	62.9 ± 3.3 **

## Supplementary Materials

The following are available online, Figure S1. Osmotic fragility curves of control erythrocytes and erythrocytes treated with 50 μM catechins. Incubation with catechins ≤5 min. Figure S2. EPR spectra of 5DS and 16DS embedded in control erythrocyte membranes and erythrocyte membranes treated with 250 μM EGCG. Figure S3. Microscopic images of control erythrocytes and erythrocytes treated with 100 μM permanganate. Human erythrocytes were analyzed using Olympus CKX53 microscope with a U-TV0.5XC-3 digital microscope camera; (a) untreated erythrocytes; erythrocytes incubated with (b) 100 μM potassium permanganate, (c) 20 μM catechin and 100 μM potassium permanganate, (d) 20 μM EGC and 100 μM potassium permanganate, (e) 20 μM EGCG and 100 μM potassium permanganate (incubation time: 5 min), (f) 20 μM EGCG and 100 μM potassium permanganate (incubation time: 20 min).
