# Supplementary material for: Interaction of Catechins with Human Erythrocytes
Source: Molecules. 2020 Mar 24;25(6):1456. doi: 10.3390/molecules25061456 (PMC7145294; doi:10.3390/molecules25061456)
Supplement: Supplementary file 1 [file molecules-25-01456-s001.pdf]

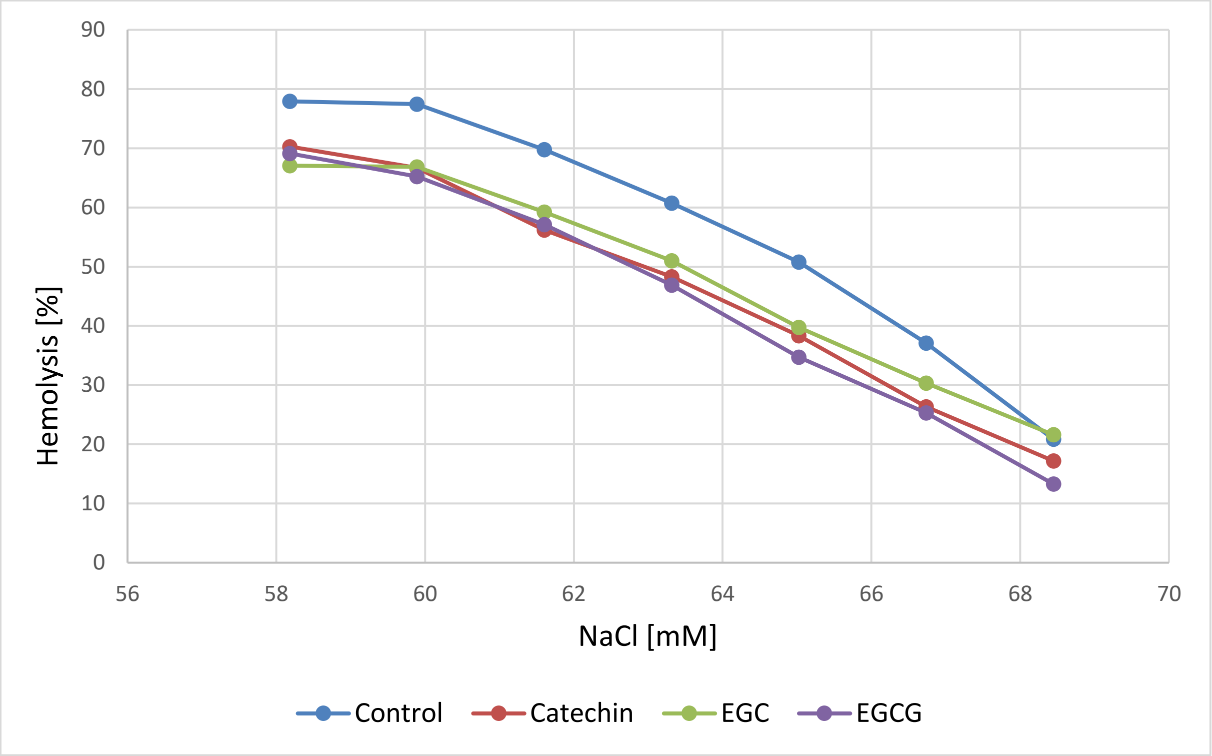

Figure S1. Osmotic fragility curves of control erythrocytes and erythrocytes treated with 50 µM catechins. Incubation with catechins ≤ 5min.

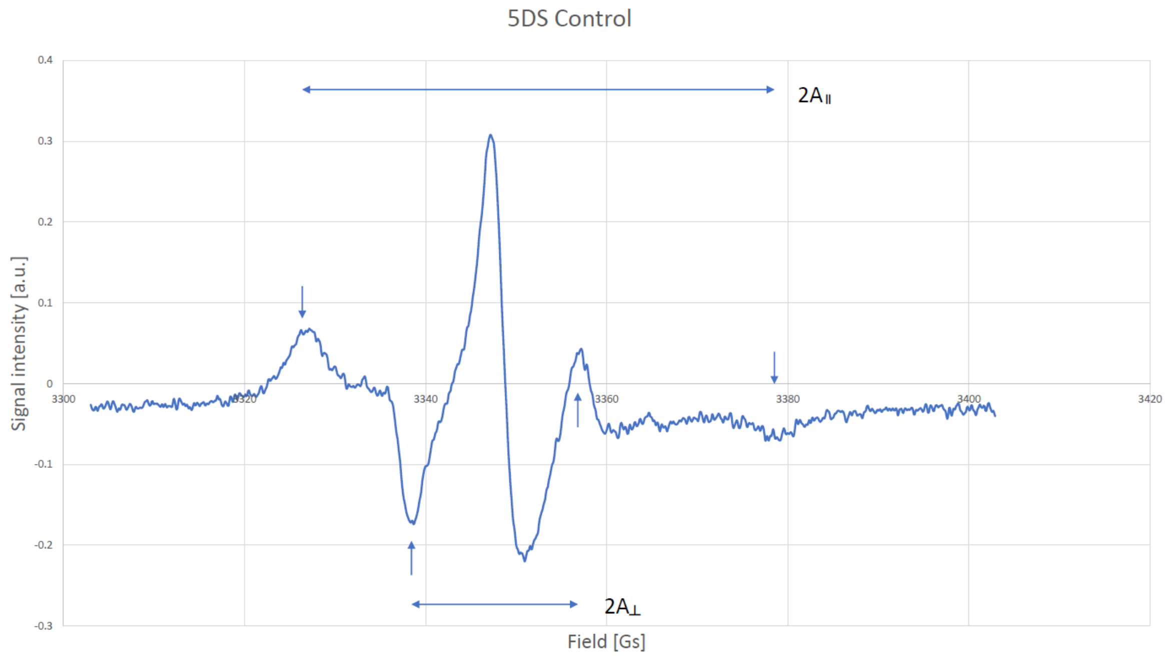

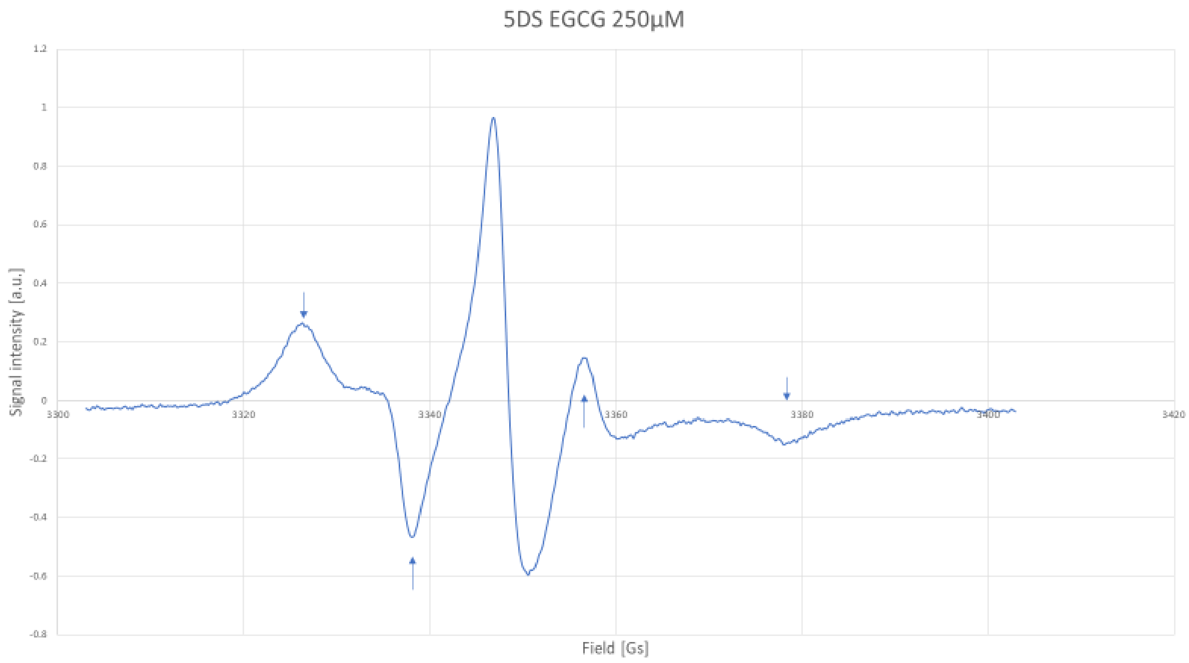
5DS EGCG 250µM
Signal intensity [a.u.]
Field [Gs]
1.2
1
0.8
0.6
0.4
0.2
0
-0.2
-0.4
-0.6
-0.8
3300
3320
3340
3360
3380
3400
3420

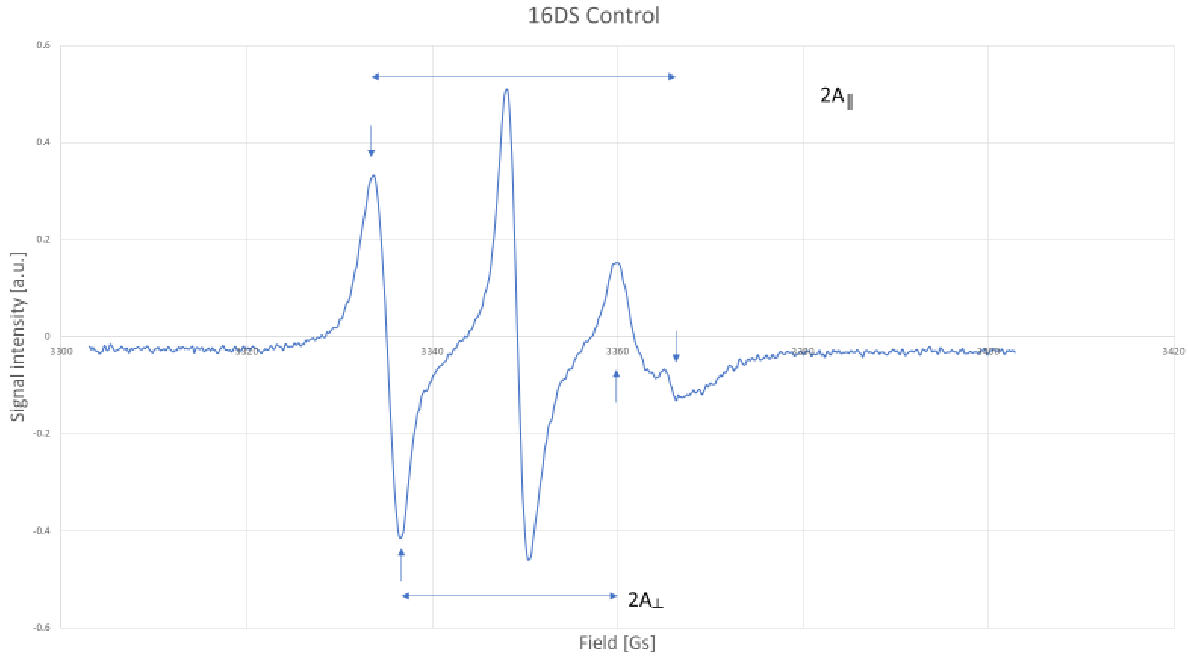
16DS Control
Signal intensity [a.u.]
Field [Gs]
$2A_{\parallel}$
$2A_{\perp}$
0.6
0.4
0.2
0
-0.2
-0.4
-0.6
3300
3340
3360
3420

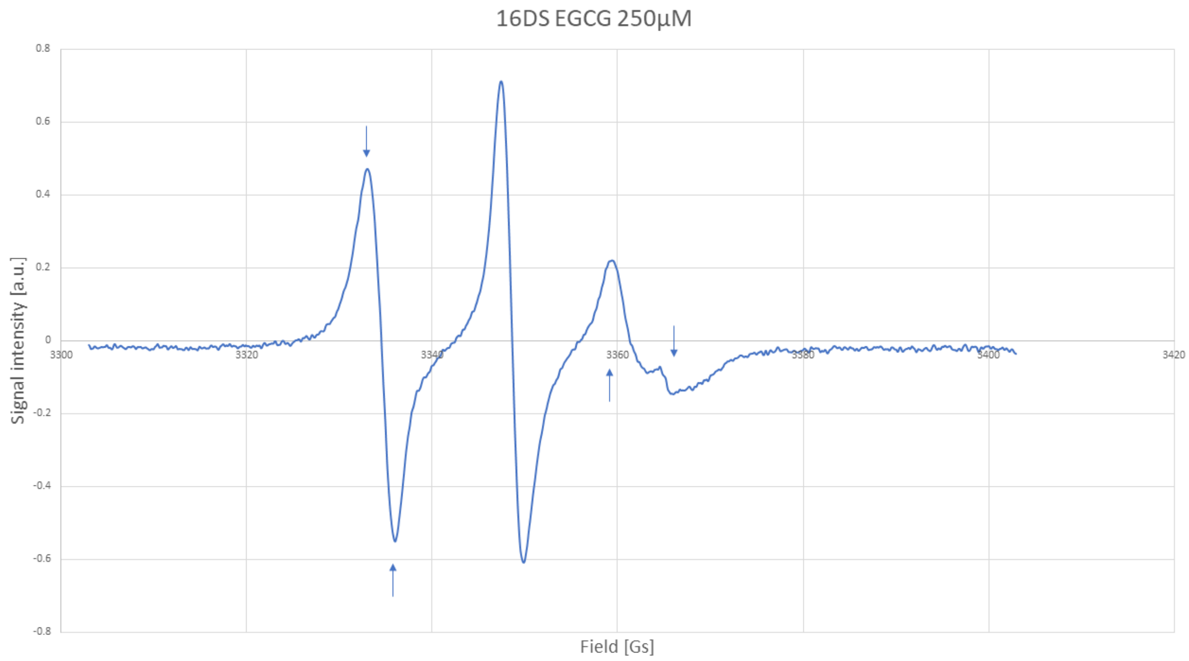

Figure S2. EPR spectra of 5DS and 16DS embedded in control erythrocyte membranes and erythrocyte membranes treated with 250 µM EGCG.

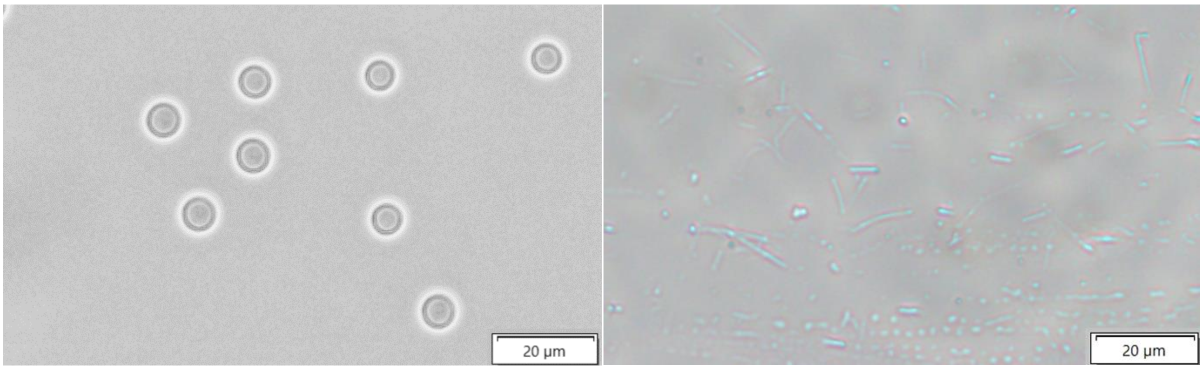

(a) (b)

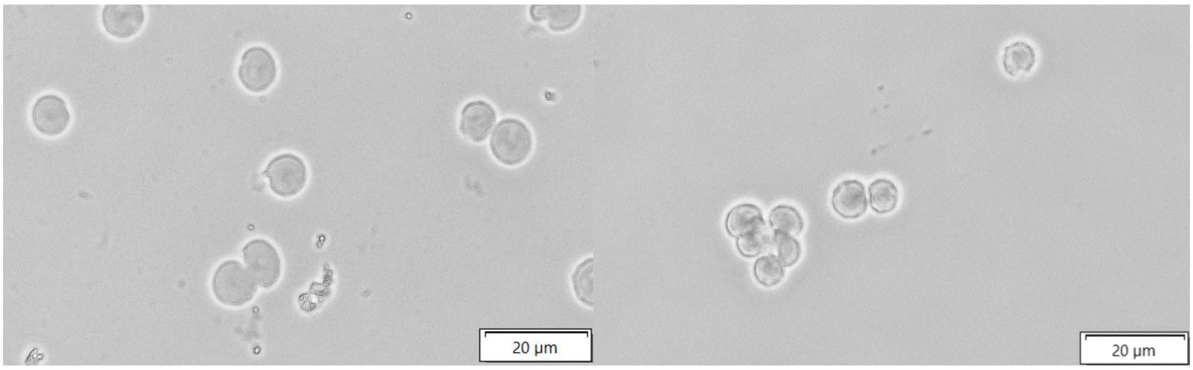

(c) (d)

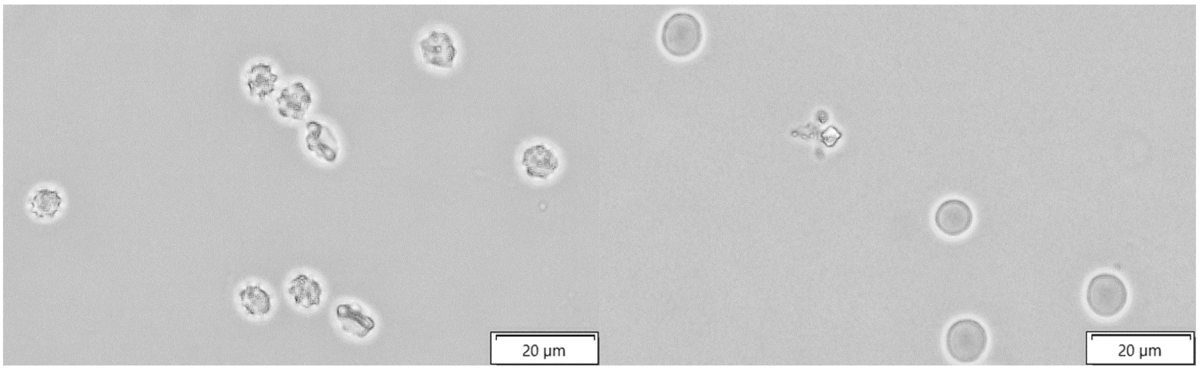

(e) (f)

Figure S3. Microscopic images of control erythrocytes and erythrocytes treated with 100 µM potassium permanganate. Human erythrocytes were analyzed using Olympus CKX53 microscope with a U-TV0.5XC-3 digital microscope camera. Images: (a) untreated erythrocytes; erythrocytes incubated with (b)100 µM potassium permanganate, (c) 20 µM (+)-catechin and 100 µM potassium permanganate, (d) 20 µM EGC and 100 µM potassium permanganate, (e) 20 µM EGCG and 100 µM potassium permanganate (incubation time: 5 min), (f) 20 µM EGCG and 100 µM potassium permanganate (incubation time: 20 min).
